# Preparation of Citral Oleogel and Antimicrobial Properties

**DOI:** 10.3390/gels9120930

**Published:** 2023-11-25

**Authors:** Shangjian Li, Jiajia Chen, Yuntong Liu, Honghao Qiu, Wei Gao, Kundian Che, Baogang Zhou, Ran Liu, Wenzhong Hu

**Affiliations:** 1 School of Pharmacy and Food Science, Zhuhai College of Science and Technology, Zhuhai 519041, China; 2 College of Life Science, Jilin University, Changchun 130015, China; 3 Zhuhai Lizhu Microsphere Technology Co., Zhuhai 519000, China; 4 College of Life Science, Dalian Minzu University, Dalian 116600, China

**Keywords:** oleogel, γ-oryzanol, β-sitosterol, citral, antibacterial coatings

## Abstract

The objective of this study was to analyze a natural and safe oleogel with antimicrobial properties that can replace animal fats while lengthening the product’s shelf life. The oleogel was created using direct dispersion (MG-SO), and its material characterization exhibited the exceptional performance of the hybrid gelant. Additionally, citral was integrated into the oil gel to prepare the citral oleogel (MG-SO). The antimicrobial nature of the material was examined and the findings revealed that it inhibited the growth of various experimental model bacteria, including *Escherichia coli*, *Staphylococcus aureus*, *Aspergillus niger*, *Botrytis cinerea*, and *Rhizopus stolonifer*. In addition, the material had a comparable inhibitory impact on airborne microorganisms. Lastly, MG-SON was utilized in plant-based meat patties and demonstrated an ability to significantly reduce the growth rate of microorganisms.

## 1. Introduction

Solid fats play a significant role in the contemporary food industry. Primarily incorporated as food additives and raw materials within food processing, they elevate the physiological and organoleptic qualities (flavors, texture, etc.) of food products [1]. It is commonly utilized in food processing, including bakery, confectionery, puffing foods, margarine [2], and meat products [3,4]. Especially in plant-based meat products, solid fat serves as an essential ingredient that replaces animal fat, and it mimics the texture, flavor, taste, and juiciness of such products. Additionally, it aids in improving their nutritional value [5,6]. Most of the solid fats currently used are hydrogenated vegetable oils and animal fats, which contain trans fatty acids in addition to high levels of saturated fatty acids. The overconsumption of these could increase the incidence of coronary heart disease, diabetes, and cancer [7]. As living standards rise, the need for food safety and nutritional health increases. Adding liquid vegetable oil directly to food can lead to several adverse effects, such as loss and leakage of fats, shortened shelf life of products, and impact on the taste and flavor. Therefore, creating a new form of healthy solid fats to substitute traditional solid fats has gained significance [8].

An oleogel is an oil-structuring system produced by adding a gelling agent to liquid edible oil [9]. In the system, the gelling agent is heated, sheared, and cooled to produce a three-dimensional network of crystalline particles, self-assembled fibers, or polymers. The liquid oil is then encapsulated in the three-dimensional network structure, causing it to lose its fluidity, and resulting in the formation of a viscoelastic semi-solid or solid grease [10]. Currently, natural gelling agents primarily comprise natural wax [11], fatty acids [12,13], cellulose [14], polysaccharides [15], lecithin, γ-oryzanol, and β-sitosterol. The gelling agents that stabilize oils by forming crystalline particles include fatty acids, fatty alcohols, monostearate [16], phytosterol, and certain natural waxes. The gels formed by these gelling agents tend to be two-dimensional or one-dimensional crystals, and a large number of small crystals are aggregated to form a large crystal network to stabilize the free oil [17]. Another gelling agent formed through self-assembled networks is sorbitol, 12-hydroxystearic acid (HAS) [18], and a mixture of γ-oryzanol and β-sitosterol. They establish a network structure with each other to create a supportive or encasing network within or outside the unbound oil to stabilize it [19].

β-Sitosterol is a naturally occurring bioactive compound of tetracyclic triterpenoids that is widely distributed in lipid-rich plants [20]. It is commonly found in plant roots, leaves, flowers, and seeds [21], and is abundant in foods such as vegetables, nuts, and vegetable oils [22,23,24]. β-Sitosterol has various pharmacological properties, including immune enhancement [25], antioxidative, and anti-inflammatory properties [26], as well as causing a reduction in cardiovascular disease [27]. β-sitosterol is a safe food additive as it has no toxic effects on the human body [28]. On the other hand, β-sitosterol’s antioxidant capacity can safeguard unsaturated oils from oxidation [29].

Citral, 3,7-dimethyl-2,6-octadien-1-al, is from a wide range of sources, naturally occurring in the leaves and fruits of many plants, and is a key component of the essential oils of many herbs such as myrtle (*Rhodomyrtus tomentosa*), lemongrass (*Cymbopogon citrates*), bee balm (*Melissaofficinalis*), and verbena (*Verbena officinalis*) [30,31]. The U.S. Food and Drug Administration (FDA) classifies citral as a food additive that is Generally Recognized as Safe (GRAS) [32]. In addition, citral itself has a highly effective broad-spectrum antibacterial ability [33]. However, like other essential oils, citral is highly reactive and volatile in the surrounding air. A series of cyclization and oxidation reactions can chemically alter citral in oxygenated environments with low pH or alkaline conditions [34,35], leading to the loss of active substances and to a reduction in its fresh lemon-like aroma. In turn, this can generate off-flavor compounds. Therefore, we used oleogel to load citral. The texture and flavor of plant-based meat pies are also enhanced using citral. The gel has the ability to safeguard and release citral [36], resulting in the preserved nutritional value of citral and an enhanced market value of the product.

Mixed gelling agents were dispersed in sunflower seed oil using a direct dispersion method, resulting in the creation of a mixed oil gel (MG-GO). Simultaneously, we evaluated glycerol monostearate gel (MG), as well as β-sitosterol and γ-oryzanol gel (SO). The effects of varying concentrations and types of gelling agent on the oleogel’s characteristics were investigated. After optimizing the formulation, the study examined the appearance, hardness, thermodynamics, crystal form, and microstructure. Finally, we evaluated the bacteriostatic efficiency of MG-SON. We developed a gel that mimics animal fat and possesses antimicrobial properties, thereby extending the shelf life of food products. This innovation presents a novel idea for the food industry.

## 2. Results and Discussion

### 2.1. Gel Characterization

#### 2.1.1. Critical Gelling Concentration, Hardness and OBC

**Figure 1:**
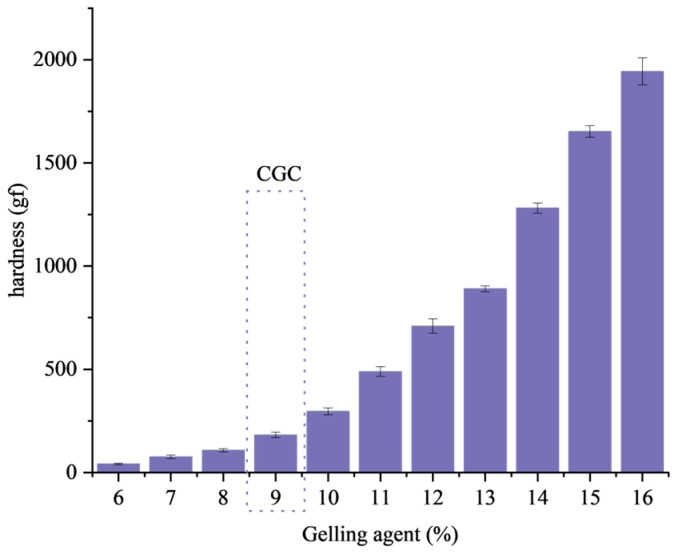
Hardness change of oleogel with the amount of gelling agent. Critical gelling concentration is labeled CGC. Error bars show the standard deviation of the mean.

The minimum concentration of gelling agent that causes the liquid oil to form a gel structure is called the critical gelling concentration, which can reflect the gelling ability of the gelling agent [37,38]. The result of the critical gelling concentration is marked in Figure 1. When the amount of mixed gelling agent is above 9%, the oil gel stops flowing and appears solid after inversion.

Hardness is an important index of gel properties, which reflects the compressive strength of gel materials [39]. The hardness results of the gel are shown in Figure 1, and the hardness of the gel increases as the amount of gelling agent increases. When the dosage of gelling agent is 9%, the oleogel no longer flows and presents a solid state with a hardness of 182 ± 12 gf. Because of the increase in gel concentration, more gels participate in the construction of the internal structure of the gel, and because β-sitosterol and γ-oryzanol self-assemble to form a crystal structure network, a high concentration of gel is more conducive to the formation of β-sitosterol and γ-oryzanol crystals. As the concentration of gelling agent increases, more crystals accumulate near the structural network, and the structure becomes more stable, thus increasing the hardness.

**Figure 2:**
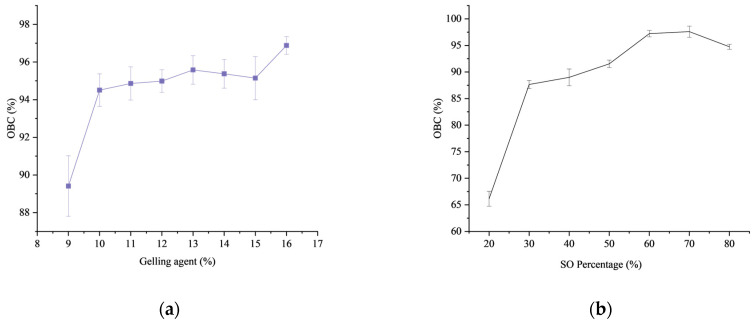
The oil binding capacity was analyzed in gels of various concentrations (**a**). Oil binding capacity results of oleogel added with 10% different composition ratio (**b**). Error bars show the standard deviation of the mean.

The oil binding capacity (OBC) reflects the ability of the internal network structure of the oleogel to retain liquid oil, which is an important index to measure the physical stability of the oleogel [40]. The correlation between the combined content of gelling agents and OBC is illustrated in Figure 2a. The figure provides evidence of increased oil gelling in OBC with an augment in the quantity of gelling agents. The size and number of crystalline units within the oil gel increases with increasing the concentration of the gelling agent. Many flaky crystals are stacked with each other to form druse and aggregate to form a stable structure, thereby improving the stability and the oil holding capacity of the oil gel. The effect of different ratios of mixed gelling agents on the oil retention capacity of the gel was investigated. As a result, as shown in Figure 2b, the oil holding capacity of the gel increased with increasing ratio of β-sitosterol and γ-oryzanol. This is because the network structure formed by β-sitosterol and γ-oryzanol is a multidimensional structure formed by itself, which is more stable than the two-dimensional crystal formed by other gels such as glycerol monostearate by many small crystals gathered to form a crystal network.

#### 2.1.2. Fourier Transform Infrared Spectroscopy (FT-IR)

**Figure 3:**
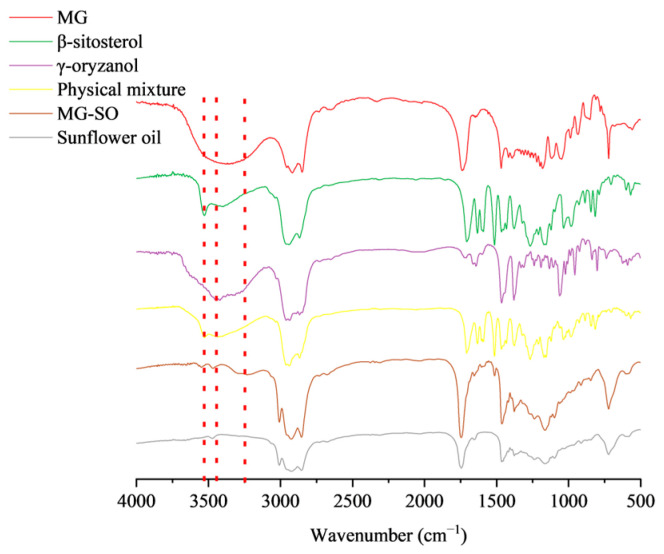
The oil binding capacity was analyzed in gels of various concentrations. Oil binding capacity results.

FTIR can be utilized for the analysis of oleogel molecular structure and component interactions [41]. FIRT results for different samples at room temperature are shown in Figure 3. It is a robust method for confirming the formation of hydrogen bonds among gel constituents [42].

The MG spectrogram is shown in Figure 3. There was a broad peak at about 3400 cm^−1^ which could be due to the stretching vibration of O-H. The -CH asymmetric stretching vibration was at 2940 cm^−1^. One of the peaks at 1738 cm^−1^ was due to the stretching vibration of C=O and is the characteristic absorption peak of stearic acid. The sunflower oil spectrum observed absorption at 1741 cm^−1^ represented the ester carbonyl stretching vibration. The β-sitosterol peak at 3528 cm^−1^ appeared to be the carboxyl group stretching vibration peak. No new peak patterns were observed in either the MG-SO or physical mixture. The shift of the carbonyl peak in the MG-SO profile towards a longer wavelength, from 1709 cm^−1^ to 1745 cm^−1^, indicated the formation of hydrogen bonds between β-sitosterol and ghrelin. These bonds resulted in the creation of a gel system through intermolecular hydrogen bonding interactions, a phenomenon that physical mixtures do not display.

The smaller shift in wave number and the change in peak width were due to the change in gel content, and no new absorption peaks appeared. The gel oil molecules were still physically entangled in the form of non-covalent bonds. Compared with other FTIR spectra, -OH absorption peak of the MG-SO sample shifted to 3248 cm^−1^, indicating that the hydrogen bond strength decreased and that the intercomponent polymer had been formed [43].

#### 2.1.3. Crystal Morphology

**Figure 4:**
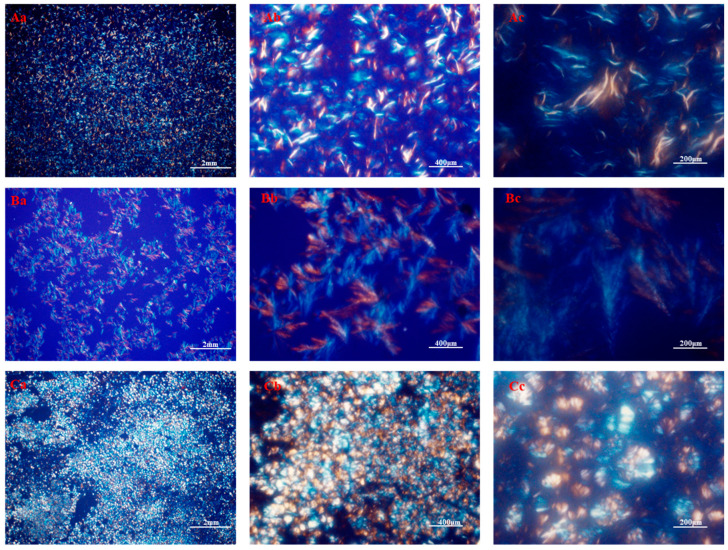
Micrographs of MG (**A**), SO (**B**), and MG-SO (**C**) obtained at 10 (**a**), 20 (**b**), and 40 (**c**) times magnification under polarized light.

After fully mixing the gelling agent and liquid oil, primary particles were precipitated or crystallized through hydrogen bonds, van der Waals’ force, and π−π stacking interactions [44]. These single primary particles then assembled into crystal particles, also known as clusters, which in turn aggregated to form larger network structures [10]. The morphology of crystals was primarily determined by the size, shape, distribution, and degree of particle aggregation. The microscopic morphology of the MG oil gel, as demonstrated in Figure 4A, displayed needle-shaped beeswax crystals that contributed to the gel structure [45]. This was attributed to the van der Waals interaction between long-chain hydrocarbons and polar ester groups on the sheet plane, leading to a high lateral growth rate that resulted in the formation of a vertical, orthogonal subcellular morphology that exhibited crystals [46]. The crystal morphology of the SO was observed using polarized light microscopy, as illustrated in Figure 4B. Apparently, SO gels formed stable oleogel structures and differed significantly from MG gels in microstructure. A dense crystalline network containing large fibrous crystals, like tree branches, was observed in the SO oleogel samples. Previous studies have also reported that molecules can self-assemble into tubular microstructures, further forming a huge crystal network that can combine the oil phase [47]. The various gelling factors crystallized autonomously to develop unique structural units that contributed to collectively enhancing the properties of the oleogel. In addition, the existence of various structural units hindered the aggregation of crystalline substances [48], thereby resulting in better spatial distribution of substances in the bulk liquid oil phase, leading to increased hardness and OBC of the oleogel. The self-assembly of gel factors through heating, melting, and stirring induced the formation of a novel molecular stacking structure in oil, resulting in the creation of a new spatial configuration. As the temperature decreased, the structure reached enhanced stability.

### 2.2. Antimicrobial Activity

#### 2.2.1. Minimum Inhibitory Concentration

The Table 1 displays the minimum inhibitory concentration (MIC) findings of citral against *S. aureus* (*Staphylococcus aureus*), *E. coli* (*Escherichia coli*), *Botrytis cinerea*, *Aspergillus niger*, and *Rhizopus stolonifer*. Citral had an inhibitory activity against both experimental bacteria and three of the experimental fungi. However, there were slight variations in the inhibitory efficacy against the five experimental microbiological. Furthermore, in numerous repetitions of the experiment, the MG-SO group exhibited no antibacterial activity. Moreover, the oil gel system itself did not possess any antibacterial properties and did not impede the evaluation of the antibacterial activity pre- and post-citral. The MIC of citral for *E. coli* was 7.812 μL/mL, which was three times greater than its MIC for *S. aureus*, which was 3.906 μL/mL. The results of this study indicate that citral has a stronger inhibitory effect on Gram-positive bacteria than Gram-negative bacteria. The MIC of citral was 15.624 μL/mL for *Botrytis cinerea* and *Rhizopus stolonifera* and 7.812 μL/mL for *Aspergillus niger*.

#### 2.2.2. Bacterial Growth

**Figure 5:**
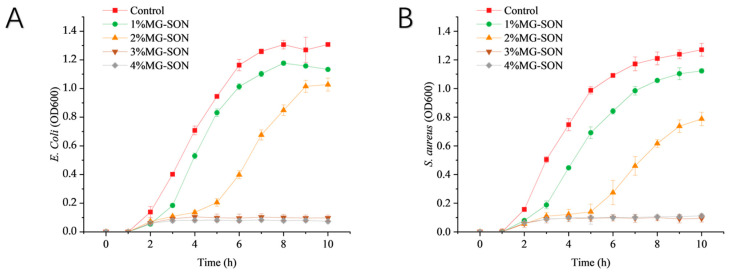
Changes in OD600 of *E. coli* (**A**) and *S. aureus* (**B**) treated with different concentrations of MG-SON during 10 h of incubation at 37 °C. 1% MG-SON is the MG-SO gel containing 1% (*v*/*v*) citral. Error bars show the standard deviation of the mean (n = 3, *p* < 0.05).

The growth curve of *E. coli* treated with MG-SON is featured in Figure 5A. It was observed that the *E. coli* in the control group attained the exponential growth phase of bacterial growth after 2 h of incubation and stabilized at 7 h. After 1% MG-SON treatment, *Escherichia coli* entered the exponential growth phase after 3 h, and entered stable phase after 8 h, and the growth curve lagged that of control group by 1 h. The stabilized OD600 values were reduced by 10.45% compared with the control group. We added 2% MG-SON treatment of *Escherichia coli* 5 h into the exponential growth phase; after 7 h the growth rate decreased and after 9 h it reached a stable phase, with the growth curve lagging behind the control group growth by 3 h. The stabilized OD600 was 20.15% less than the control group.

The growth curves of *S. aureus* treated with MG-SON are displayed in Figure 5B. The outcomes demonstrated that the growth of *S. aureus* in the control group entered the stabilization phase at 9 h after reaching the exponential growth phase after 2 h. After 1% MG-SON treatment, *S. aureus* entered the exponential growth phase after 3 h and stabilized after 10 h. The growth curve lagged behind the control by 1 h. After 2% treatment, *S. aureus* entered the exponential growth phase after 5 h, and the growth rate declined after 8 h. The growth rate reached the stabilized phase after 9 h. The growth curve lagged behind the control by 3 h. The growth curve lagged behind the control by 3 h. The 1% and 2% concentrations of MG-SON-treated *S. aureus* exhibited reductions of 11.37% and 36.07%, respectively, in OD600 compared with the control. The MG-SON with percentage concentrations was ineffective at inhibiting the growth of both *E. coli* and *S. aureus*. The percentage concentration was exceeded by the inhibitory concentration owing to the encapsulated state of citral in the MG-SON gel. The released guest substance acted slowly on the external environment. The initial exposure of microbiological to citral occured at concentrations lower than the doctrinal concentration, leading to ineffective bacterial inhibition. The release characteristics were similar to a chitosan gel study by Gao Y et al. [49].

#### 2.2.3. Fungal Growth

**Figure 6:**
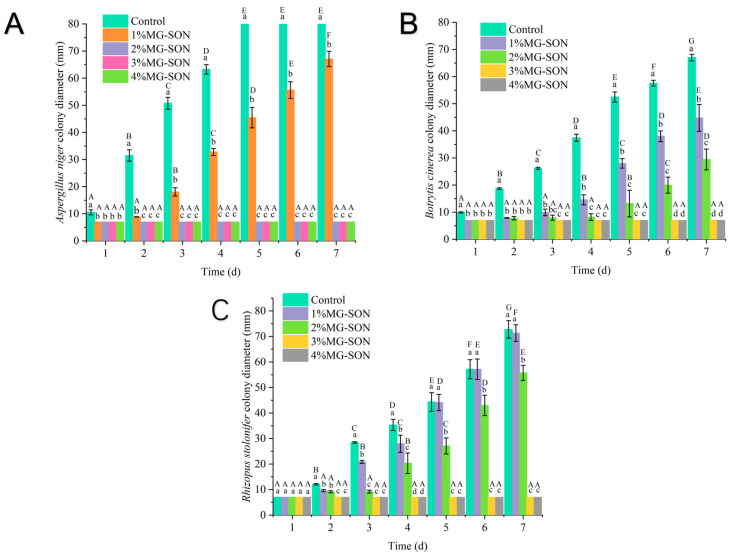
Results of alterations in colony size of *Aspergillus niger* (**A**), *Botrytis cinerea* (**B**), and *Rhizopus stolonifer* (**C**) during incubation for 7 days with varying concentrations of MG-SON medication. Where 1% MG-SON is the MG-SO gel containing 1% (*v*/*v*) citral. Error bars show the standard deviation of the mean. Different lowercase letters indicate significant differences between samples of different treatment groups with the same storage time, and different capital letters indicate significant differences between samples of the same treatment with varying storage times (n = 3, *p* < 0.05).

**Figure 7:**
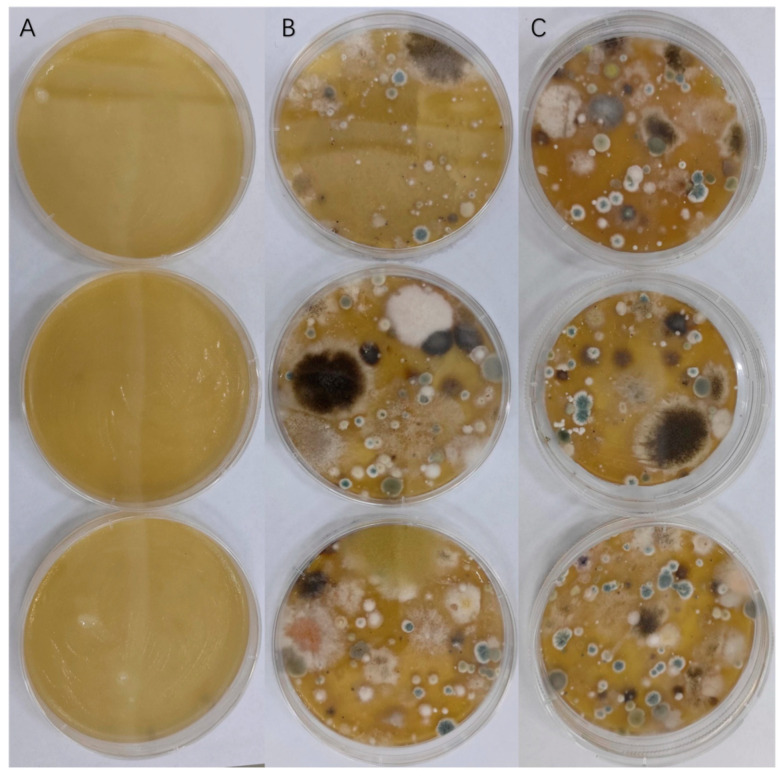
(**A**) is a PDA plate coated with 4% MG-SON, (**B**) is a PDA plate coated with MG-SO, and (**C**) is a blank control. Where 4% MG-SON is the MG-SO gel containing 4% (*v*/*v*) citral. The room temperature at the time of the experiment was 26 °C with 68% humidity.

The fungal culture findings are displayed in Figure 6. As Figure 6A illustrates, the growth of *Aspergillus niger* within a 7-day incubation indicates that it was incapable of growing on a PDA medium coated with concentrations of MG-SON greater than 2%. Additionally, after incubation for 7 days at the 1% MG-SON treatment, the growth diameter of *Aspergillus niger* colonies was significantly lower than that of the control group (*p* < 0.05). *Botrytis cinerea* growth is illustrated in Figure 6B, and it evidences an incapacity to thrive at concentrations of 2% or higher. *Rhizopus stolonifer* growth is illustrated in Figure 7C, and it evidences an incapacity to thrive at concentrations of 3% or higher. The experimental results agreed with the results of a citral liposome inhibition by Chen et al. [50]. Citral can inhibit the growth of microorganisms well.

#### 2.2.4. Airborne Microorganisms

Numerous airborne microorganisms pose a threat to our food supply. The results indicate that the 4% MG-SON-coated PDA medium exhibited no substantial microbial growth or multiplication. Figure 7 displays the outcomes of MG-SON effectiveness against these microorganisms. Contrastingly, the PDA plates of the MG-SO and blank control group were teeming with microorganisms. Citral was loaded onto MG-SO, which facilitated citral’s antimicrobial effect and prolonged its action. Additionally, MG-SO acted as a protective agent for citral. The study found that 4% MG-SO exhibited an effective antimicrobial activity for up to 7 days. Additionally, the PDA medium in the treatment groups MG-SON and MG-SO did not undergo rehydration, while the PDA medium in the control group underwent dehydration. This occurrence may be linked to the gel coating on the surface of the PDA medium, which effectively inhibited the evaporation of moisture [51].

#### 2.2.5. Microbiological Analysis of Plant-Based Meat

**Figure 8:**
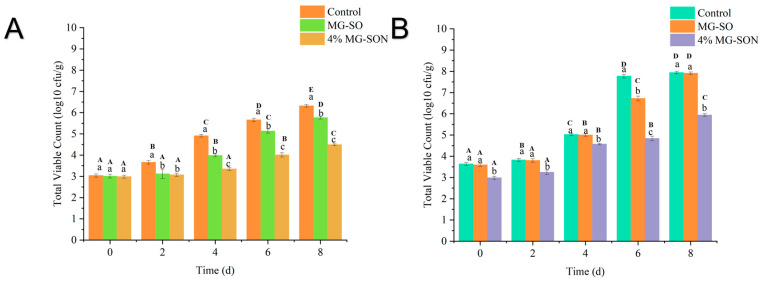
Total bacterial counts in different plant-based meat patties at 4 °C (**A**) and 25 °C (**B**) for 8 days. Where 4% MG-SON is a plant-based meat containing 4% MG-SON. Error bars show the standard deviation of the mean. The same lowercase letters indicate significant differences between samples of different treatment groups with the same storage time, and different capital letters indicate significant differences between samples of the same treatment with varying storage times (n = 3, *p* < 0.05).

The results of changes in the Total bacterial counts in plant-based meat patties in meat patties over 8 days are shown in Figure 8. During the 0–8 day storage period, MG-SON was found to inhibit the growth of the total bacterial counts. The inclusion of MG-SON in the meat patties resulted in a significant decrease (*p* > 0.05) in total bacterial counts compared with the control group. The findings were comparable to research on how a coating of alginate complexed with essential oil from lemongrass could effectively hinder the total bacterial growth on fresh pineapple cuts [52]. This demonstrated the excellent antimicrobial properties of MG-SON-loaded citral. Interestingly, the MG-SO unloaded with citral showed a reduction in the total microorganism count compared with the control group. This result could be attributed to the oxygen isolation by the gel to achieve a similar effect to that of modified atmosphere packaging for the preservation of freshness [53].

## 3. Conclusions

In this study, a natural gel-like antimicrobial coating was prepared with the aim of improving the shelf life of food products. This will contribute to preserving food products. In this paper, a simple characterization of the gel material is carried out, along with testing the antimicrobial capacity of the material and applying it to plant-based meat patties. The addition of a 10% mixed gelling agent resulted in the transformation of sunflower oil into a gel with a hardness of 296 ± 16 gf and an oil binding capacity of 94 ± 0.86%, and it can mimic the hardness of animal fats well. The application of mixed gelling agents under polarized light microscopy revealed a distinctive network of spherical crystals. Additionally, the denser structure of the oleogel enhances its capacity for holding oil, which ultimately improves the stability of the added guest substance. The loading of citral allows the oil gel to be added into the plant-based meat so that it can mimic animal fat while exerting its antimicrobial capacity. In an antimicrobial assay conducted in vitro, the growth of *E.Coli* and *S. aureus* after 10 h using 3% MG-SON, along with *Aspergillus niger*, *Botrytis cinerea*, and *Rhizopus stolonifer*, was inhibited within a period of 7 days using 3% MG-SON. MG-SON has been demonstrated to effectively decrease the overall bacterial count in plant-based meat patties while in storage.

## 4. Materials and Methods

### 4.1. Materials

#### 4.1.1. Reagents

The sunflower seed oil (MIGHTY), β-Sitosterol (95%, CAS: 83-46-5, Shanghai Yuanye Biotechnology Co., Ltd., Shanghai, China), γ-Oryzanol (98%, CAS: 11042-64-1, Shanghai Yuanye Biotechnology Co., Ltd., Shanghai, China), Monostearin (AR, CAS: 123-94-4, Shanghai Yuanye Bio-Technology Co., Ltd., Shanghai, China), Citral (97%, CAS:472-61-7, Macklin, Shanghai, China), and plant-based meat patties were provided by the Pharmacy and Food Science Laboratory of Zhuhai University of Science and Technology. The ingredients (soy protein, pea protein, gluten meal, carrageenan, inulin, and starch acetate); Potato Dextrose Agar (PDA) (021052, HKM); Tryptic Soy Broth (TSB) (024051, HKM, Guangzhou, China).

#### 4.1.2. Strain Information

*Escherichia coli* (*E. Coli*) (GDMCC, 1.1917); *Staphylococcus aureus* (*S. aureus*) (GDMCC, 1.2636); *Aspergillus niger* (CICC, 2089); *Botrytis cinerea* (CGMCC3.3789); *Rhizopus stolonifer* (CGMCC, 3.31).

### 4.2. Instruments and Equipmen

Scanning Electron Microscope (TESCAN MIRA, TESCAN, Brno, Czech Republic); UV Spectrophotometer (Shimadzu, UV-2600i, Japan); frozen centrifuges (1248R, Genecompany, Hong Kong, China); Differential Scanning Calorimeter (DSC 214 Polyma, Netzsch, Germany); Fourier Transform infrared spectroscopy (Shimadzu, IRAffinity-1S, Japan); X-ray Diffractometers (Bruker, D8 DISCOVER, Germany); Texture analyzer (Bosin, TA. TOUCH, China); Handy plate^®^ Aerobic Count Plate (Huankai, Handy plate^®^, China).

### 4.3. Gel Characterization

#### 4.3.1. Critical Gelling Concentration

Taking 100 g of sunflower oil, 6%, 7%, 8%, 9%, 10%, 11%, 12%, 13%, 14%, 15%, and 16% (*w*/*w*) of the gelling agent was added, separately. The mixture was heated and agitated in a water bath at 90 °C for 60 min. Subsequently, it was removed from the bath. Then, 1% citral was added and mixed well, before refrigerating it at 4 °C overnight to procure an oil gel. The centrifuge tube was inverted and filled with oil gel, its flow was observed, and the state of gel formation was assessed; the ability to flow was used as the basis for judgment [54]. The critical gelation concentration was then accurately determined.

#### 4.3.2. Oil Binding Capacity (OBC)

The OBC was determined by centrifugation [55]. The mass of the empty centrifuge tube was recorded as $m_{1}$. We took 1 mL of molten oleogel and placed it in the empty centrifuge tube. It was left in the refrigerator at 4 °C overnight to form the oleogel. Then, it was stored at 25 °C. The mass after 2 days of storage was recorded as $m_{2}$. The sample was placed in a centrifuge and centrifuged at 10,000× *g* rpm for 10 min. Then, it was taken out. After inverting the sample to drain the precipitated liquid oil, in was weighed and its mass was recorded as $m_{3}$. The oil retention rate was calculated from the formula.

$$OBC\left(\%\right)=\frac{\left(m_{2}-m_{1})-(m_{2}-m_{3}\right)}{m_{2}-m_{1}}$$

where $m_{1}$ is the mass of the empty centrifuge tube, $m_{2}$ is the total mass of the centrifuge tube and oleogel, and $m_{3}$ is the total mass of the centrifuge tube and oleogel after centrifugal discharge of the liquid oil.

#### 4.3.3. Hardness

The hardness of the oil gel samples was measured using a texture analyzer. It was refrigerated overnight at 4 °C. The P20 probe was selected, and the measurement parameters were as follows: The pre-test speed was 0.5 mm/s, the test speed was 0.5 mm/s, the post-test speed was 2 mm/s, the compression degree was 70%, the trigger force was 10 g, the sample was parallel five times and the average value was taken, and the maximum force of pressure was defined as the hardness value of the gel.

#### 4.3.4. Fourier Transform Infrared Spectroscopy (FT-IR)

The oleogel infrared spectrum was determined using a FT-IR spectrophotometer at a room temperature of 27 °C. The spectral scan range was from 4000 to 500 cm^−1^.

#### 4.3.5. Crystal Morphology

Microscopy was utilized to study the crystalline morphology of the oleogel under polarized light [56]. The oil gel samples were positioned on glass slides, covered with coverslips, and stored at 4 °C for 24 h before being brought under the microscope. The observation was conducted using objective lenses of 4, 20, and 40×.

### 4.4. Antimicrobial Activity

#### 4.4.1. Minimum Inhibitory Concentration

To determine the minimum inhibitory concentration (MIC) of citral using 96-well plate zymography, we first added 100 μL of TSB medium mixed with TTC to each well of the 96-well plate. Starting from the first well, 100 μL of citral was added to each well, sequentially, until the 12th well. Then, 100 μL of the mixture was disgarded from the final well. Then, 100 μL of bacterial solution (1 × 10^6^ CFU/mL) was added to each well. The wells were cultured at 37 °C. Each row of 12 wells comprised an experimental group, with the blank group consisting of the culture medium and the control group consisting of the TSB medium. Each of the experimental bacteria were tested three times to ensure accuracy. PDW medium was used for the fungal culture.

#### 4.4.2. In Vitro Antibacterial

To culture bacteria using this method, 100 mL of TSB medium was added to a shake flask. Then, the appropriate amount of MG-SON was added to the bottom of the TSB culture medium based on the 1%, 2%, 3%, and 4% levels. The shake flask was inoculated with 6 mL of culture medium containing a concentration of 1 × 10^6^ CFU/mL and was incubated at 37 °C while shaking at 300 rpm/min. The OD600 values were recorded every hour. The experiment was repeated with five biological replicates per group.

#### 4.4.3. In Vitro Antifungal

Fungal growth was assessed by recording the colony diameter. Each fungus was inoculated with PDA medium containing varying concentrations of MG-SON before measuring the daily colony diameter for seven days, with three replicates per group.

#### 4.4.4. Resistant to Airborne Microorganisms

The PDA plates coated with 4% MG-SON and MG-SO were observed for microbial growth after being left open and exposed to air for 7 days. The blank group consisted of untreated PDA plates, and each group was repeated three times.

#### 4.4.5. Microbiological Analysis of Plant-Based Meat

Plant-based meatloaf with 10 g of corn oil was included in the blank group, while plant-based meatloaf with 10 g of MG-SO was used as the control group. The experimental group consisted of plant-based meat patties with 10 g of 4% MG-SON. All three groups of meat were stored at both 4 °C and room temperature of 25 °C with plastic wrap covering. The total bacterial counts were measured every two days and the experiment was repeated three times for each group. The suitable count range was 30 cfu to 300 cfu. Counting was performed using the Handy plate^®^ Aerobic counting plate.

## 5. Statistical Analysis

The experiments were performed three times and the mean and standard deviation were obtained for each experiment. Data were analyzed using IBM SPSS Statistics software (version 26.0; IBM, Chicago, IL, USA). Data were analyzed for differences using ANOVA and the mean of each group was compared using Duncan’s multiple range test at the significance level described above; data were expressed as mean ± standard deviation. The level of statistical significance was set at *p* < 0.05.

## Figures and Tables

**Table 1:** Minimum inhibitory concentration of citral.

Strain Variety		*S. aureus*	*E. coli*	*Botrytis cinerea*	*Aspergillus niger*	*Rhizopus stolonifer*
MIC (μL/mL)	MG-SO	-	-	-	-	-
	Citral	3.906	7.812	15.624	7.812	15.624

“-” indicates no inhibition.

## Data Availability

All data and materials are available on request from the corresponding author. The data are not publicly available due to ongoing researches using a part of the data.
